# Communication Needs for Individuals With Rare Diseases Within and Around the Healthcare System of Northern Ireland

**DOI:** 10.3389/fpubh.2019.00236

**Published:** 2019-08-21

**Authors:** Ashleen L. Crowe, Amy Jayne McKnight, Helen McAneney

**Affiliations:** Centre for Public Health, School of Medicine, Dentistry and Biomedical Sciences, Queen's University Belfast, Belfast, United Kingdom

**Keywords:** rare disease, communication, information systems, patients, healthcare, public awareness

## Abstract

**Objective:** By definition a rare disease affects fewer than 1 in 2,000 people but collectively 1 in 17 people are affected at some time in their lives. Rare disease patients often describe feeling isolated and unsupported. The needs of individuals living with rare disease(s) are not well met globally and have not been specifically explored in Northern Ireland.

**Methods:** An online survey was conducted in spring of 2017, focused on information and communication needs, to identify overarching themes. Databases were searched to place responses in an international context.

**Results:** There were 240 survey respondents with four overarching themes identified: sources of information; medical care; rare disease community; and public awareness. Thirty relevant papers resulted from the literature search. A coordinated and transparent approach for improved medical care is needed where researchers, practitioners, and policy makers work with patients, carers, and rare disease advocates to ensure a fully considered rare disease strategy is implemented. In line with that developed by many other countries, a physical or virtual Northern Ireland reference network or center of excellence for rare diseases would provide an important strategic link. Sustainable funding, resources for rare disease charities, and more cross-border working would help build a local rare disease community. Major challenges highlighted include finding the right health and social care information. The internet was the most regularly accessed, and perceived as the easiest way, to source information on rare disease. Improved signposting to accredited information, ideally by the creation of a locally relevant online information hub, a local rare disease registry that can integrate with international systems, a local rare disease coordinator, and improving public awareness are urgent needs.

**Conclusions:** Aligned to internationally reported outcomes, practical issues for future development based on the voices of individuals living, and working with a rare condition are described. It is essential that ongoing research evaluates changes to ensure that the best possible structures and mechanisms are put in place to improve communication and information systems for those affected by a rare condition(s).

## Introduction

A rare disease is defined as occurring in <1 in 2,000 people (1) and is often associated with major unmet health needs (2). There are more than 8,000 rare diseases identified and cumulatively rare diseases are common, representing a significant public health concern. One in 17 people in the United Kingdom (UK) being affected at some time in their lives (3). Problems encountered because of the low incidence of individual rare diseases include lack of accurate diagnosis, lack of clarity about which specialist referrals are required post-diagnosis, lack of coordinated clinical approach, insufficient social support, and medical professionals who have not heard of a disease but are treating affected patients (3). This makes it difficult to optimize treatment strategies that effectively manage the rare disease, and to know what treatment options are most appropriate across specialities. Therefore, improving communication mechanisms both within, and around the healthcare system is of vital importance to individuals living and working with rare diseases.

In 2009 the European Council recommended that member states should have in place a plan or strategy for rare disease to integrate local, regional, and national developments in this field (2009/C 151/02). This prompted a UK-wide consultation toward a UK Strategy for Rare Disease, which was published in 2013 with approval from Health Ministers in Scotland, Wales, England, and Northern Ireland (3). A Northern Ireland Rare Disease Stakeholder Forum (including representatives from the Department of Health and Social Services, Health and Social Care Board, Public Health Agency, clinicians, service users, advocacy groups, and researchers) developed a statement of intent for Rare Diseases in Northern Ireland (2014), leading to the publication of the first Northern Ireland Rare Diseases Implementation Plan (4). This Northern Ireland Rare Disease Implementation Plan was informed by the stakeholder forum and aligned to the Northern Ireland Executive's Programme for Government and “Transforming Your Care” policy which described a new model for the delivery of integrated health and social care services in Northern Ireland. Northern Ireland does not have a center of expertise for rare disease, is not a European reference network, does not have a rare disease coordinator, does not have a rare disease national/regional office, and does not have a national rare disease registry. A not-for-profit charity was established in 2012 to help connect, educate, innovate, and advocate for individuals living, and working with rare disease(s)—The Northern Ireland Rare Disease Partnership (www.nirdp.org.uk). The Northern Ireland Rare Disease Implementation Plan incorporates six key themes describing 51 commitments, (1) empowering those affected by rare disease, (2) identifying and preventing rare disease, (3) diagnosis and early intervention, (4) coordination of care, (5) the role of research, and (6) cross-border collaboration with the Republic of Ireland. The research presented in this manuscript will inform all themes in this Northern Ireland Plan.

The aim of this research is to evaluate the perceived availability of resources and communication preferences across Northern Ireland, and to place these findings in the wider international context by conducting a comprehensive literature review.

## Methods

### Survey

A survey was developed in collaboration with the Northern Ireland Rare Disease Partnership (NIRDP), which is a central organization in Northern Ireland aiming to advocate, connect, educate and innovate for individuals living and working with rare diseases across Northern Ireland. NIRDP is a not-for-profit partnership of individuals living and working with rare diseases in Northern Ireland including researchers, clinicians, allied health professionals, patients, carers, voluntary groups, rare disease charities, and industry partners. This survey aimed to evaluate communication for rare diseases within the healthcare system, how individuals' access or engage with medical practitioners, and how individuals generally access information about rare diseases. The target audience was any individual living and/or working with a rare disease in Northern Ireland and there is ethical approval from research ethics committee, Northern Ireland. Demographic information was collected and stored separately using the following questions:

- Do you currently live in Northern Ireland?
- What is your gender?
- Which age bracket best describes you?
- Which group best describes your ethnic background or association?

Also included were questions to help prioritize improvement within, and allied with, communication and information processes of the integrated health, and social care system in Northern Ireland.

The online survey was promoted by the NIRDP *via* their social media accounts on Twitter (@NI_RDP; 1,090 followers) and Facebook (@NIRDPNews; 858 members) from January-June 2017 and further disseminated by individuals, member charities, and voluntary groups; there was not a mechanism available to track the reach of this dissemination through social media. The survey was also described on the NIRDP website with a lay summary (https://www.nirdp.org.uk/) and at four public rare disease meetings geographically spread across Northern Ireland in March and April 2017, where there was also the opportunity to complete the survey in hard copy. Informed consent was obtained from all participants. Consent was explicitly given by each participant by actively ticking a “consent box” before they could proceed with the survey. Anonymized responses were collated in a spreadsheet and appropriately summarized in tables and charts; respondents had the option to skip questions without answering. Questions were of various formats including tick-box, ranked and open text narrative responses (see Online Resource 1 for wording). There was the option to comment after all but one of the questions and these comments were considered as part of the results. Seven questions related to existing information, two to improving information, and communication services in the future, and one question asked for a general response to the overall theme. Data retrieved from the information and communication survey were analyzed both quantitatively and thematically.

### Literature Review

**Figure 1:**
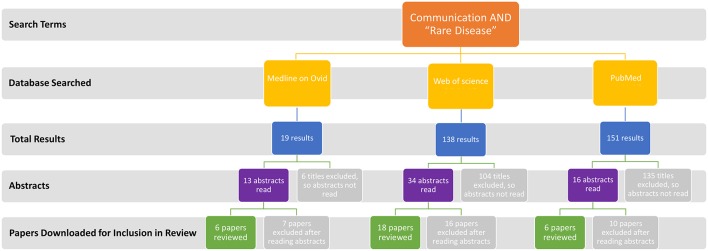
Flow chart illustrating literature review process where 30 full text articles were included in the final analysis. Papers were excluded after review of abstracts as they were: not focused on rare disease; not to do with the topic of communication; about medical issues directly rather than the communication around them; not in English; published prior to 2012; to do with animals rather than humans; not relevant to person to person communication (genetic communication for example); about research practice rather than how to access research; related to achieving diagnosis rather than what is communicated around it; about symptoms which make communication difficult rather than communication about the rare disease.

A comprehensive review of literature published since 2012 and written in English was conducted. Resources MEDLINE on Ovid, Web of Science, and PubMed were searched using search terms “communication” AND “rare disease,” with and without including “Northern Ireland” (last updated 11th January 2018). Papers were initially screened by title with duplicates removed, and then screened by abstract. The remaining articles were screened by reading the full text and all those apparently relating to communication in people living and working with rare disease were included; no specific type of study or study population was excluded at this stage. Papers were excluded after review of abstracts if they were: not focused on rare disease; not to do with the topic of communication; about medical issues directly rather than the communication around them; not in English; published prior to 2012; to do with animals rather than humans; not relevant to person to person communication (genetic communication for example); about research practice rather than how to access research; related to achieving diagnosis rather than what is communicated around it; about symptoms which make communication difficult rather than communication about the rare disease (Figure 1). For those articles included in the review, the following data were extracted: year of publication, country of study, and main findings (see Online Resource 2).

## Results

### Survey Participants

**Figure 2:**
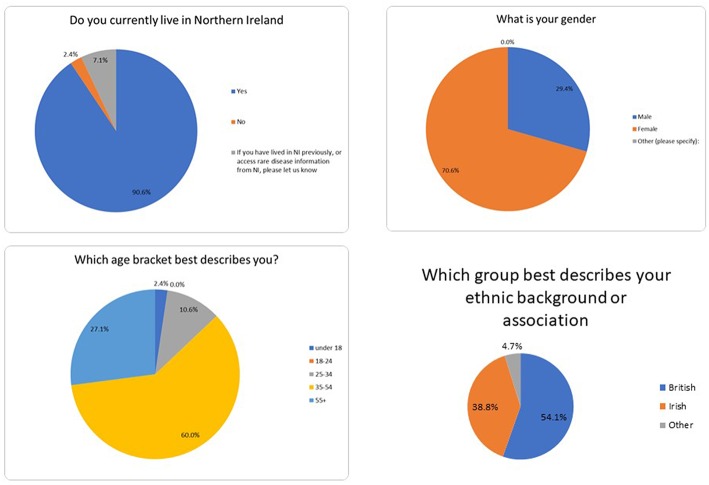
Sociodemographic information from respondents.

240 participants completed the survey, with 38% (91/240) completing the survey in full. Thirty one percent (75/240) respondents identified themselves as a patient living with a rare disease, 19% (45/240) as family of a person with a rare disease, 18% (44/240) as carers and 14% (33/240) as healthcare professionals. Others who completed the survey identified as medical doctors, social support practitioners, managers, policy makers, researchers, volunteers for rare disease charities, and staff working in the charity sector. Interestingly, 22 people identified as both carers and family members, indicating that people were reporting with experience of multiple roles. There were 29% male and 71% female respondents who described a gender, plus 6 respondents who did not specify. The majority of respondents (87%) were over the age of 35 years, with 93% identifying as British or Irish ethnicity (Figure 2).

### Survey Responses for Information and Communication

**Figure 3:**
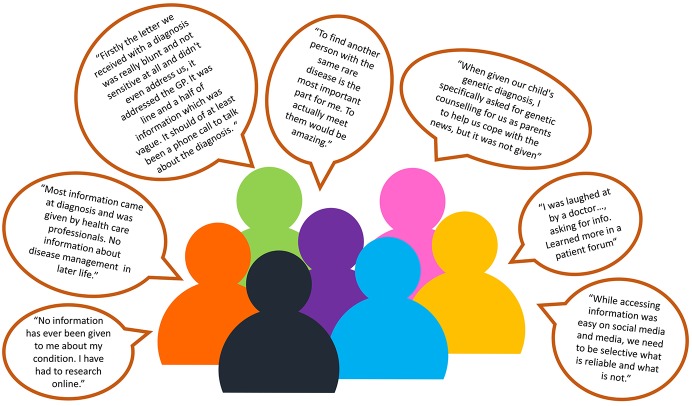
Quotes from participants in the information and communication survey demonstrating the voice of the rare disease community in Northern Ireland.

**Figure 4:**
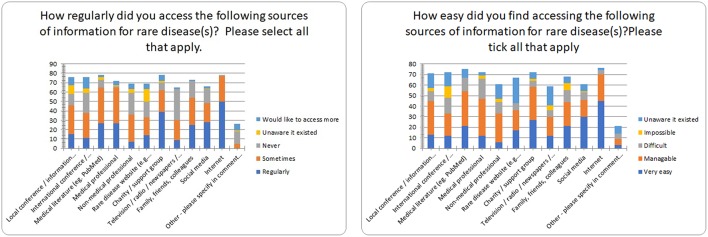
Regularity and ease of access of information sources for rare disease.

This survey resulted in a breadth of highly emotive responses (Figure 3). The internet was the most regularly accessed source of information for rare diseases (65% accessed it regularly) followed by charity/support groups (48%). Results illustrating how regularly information sources were accessed are summarized in Figure 4. Orphanet, conferences, and information events focused on rare disease were the three sources of rare disease information where respondents most wished to know more. Respondents reported varying levels of ease accessing diverse information sources with only 78 individuals completing this question in detail (Figure 4). Fifty eight percent of respondents found the internet very easy to access and 32% manageable as an accessible source of information, followed by social media with 38% stating it was very easy, and 21% manageable for access. While access was relatively easy and 97% of respondents found the internet useful, respondents noted that they often did not know what was reliable when they were reading it online. Half of respondents struggled to access information on conferences with more respondents finding it harder to access information from medical professionals (32%) than from medical literature (27%).

**Figure 5:**
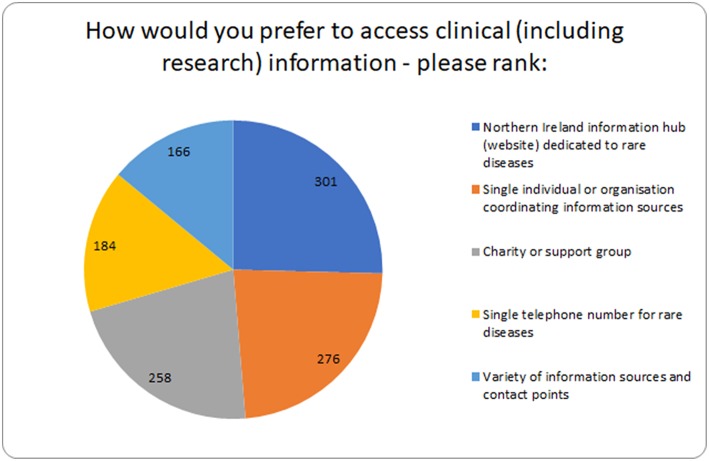
Summary of how individuals prefer to access clinical and research information.

The preferred top-ranked mechanism by which respondents would wish to obtain clinical or research information in the future was *via* a Northern Ireland information hub (website) dedicated to rare diseases (Figure 5). A single individual or organization coordinating information sources, and from a charity or support group were second, and third ranked respectively. Several respondents commented they were keen to have access to a Center of Excellence (locally or overseas), or international resource to obtain informed advice from experts, while others would prefer a multidisciplinary hub connecting hospital professionals directly with primary care physicians and community medical professionals. One hundred and eight individuals stated a preference for receiving face to face information and support via disease specific charity events, with tailored advice also available online, and in hard copy.

Two responses for the quality and quantity of information were very positive commenting, “*I have all the information that I need in relation to managing…”* and “*the NHS website provides useful templates about disease specific information*,” however this was unusual. Most respondents described wanting more information regarding diagnosis, prognosis, individual care plans, relevant clinical trials/therapies, counseling, clinical psychological support, improving lifestyle, and day to day living. More than ¼ of respondents wanted more information at diagnosis, while 30% of respondents were seeking more clinical information on treatments. The majority of patients wanted access to their own medical records as they are the coordinators of their often-complex care needs.

In terms of non-clinical support, peer support was the most commonly accessed (61%), with financial support (49%), educational support (40%), and family support (39%) also being frequently used. One respondent described their diagnosis as, “*a life-changing event*.” Non-clinical psychological support/counseling had the most responses of being unable to find information regarding it (11%; 28% never tried to access), relative to other non-clinical support options. Several respondents commented they advocate and or coordinate their own social care support.

### Survey Responses for Engagement and Priorities for Change

Several suggestions were described to promote good engagement with individuals and/or teams involved with rare diseases, including:

▪ A rare disease coordinator, although views varied on the role of such a coordinator. Some individuals asked for a coordinator whose role is to provide access to a variety of services using a joined up multi-disciplinary approach, while others requested a coordinator with a more managerial role signposting to relevant information and resources.
▪ “*Good communication skills with the family and carers to enable a degree of advanced planning when looking at a disease trajectory or specific symptom led need*.”
▪ Improved medical resources with a “joined up” approach to rare diseases, more support, more specialist clinical staff, regional clinics, more GP education on rare diseases, more local information sessions for professionals.
▪ Reference network(s) enabling peer support (patients, family members, carers, healthcare professionals) to connect.
▪ Working in an effective partnership with a coordinated approach. “*Accessibility …& mutual respect…combined with openness and willingness to listen…reach consensus agreement on actions to be taken… and act.”*
▪ Clear communication with good information and signposting. More public meetings, rare disease community consultations, and social media engagement.

Barriers to engagement centered around poor communication, insufficient resources, lack of transparency, lack of respect for the patient voice, lack of local rare disease registry/poor identification of individuals with a rare disease, lack of awareness, and understanding, geographical separation/physical inaccessible, and not knowing where or how to access support, services, and information. Survey respondents indicated a serious problem for a person with a rare disease is that health and social care professionals don't listen with respondents wanting more “*involvement of patients as experts,”* “*empathy*,” and “*a more open mind from consultants when we present information we have found*.”

A range of priorities were suggested to improve information sharing and communication for rare disease(s) in Northern Ireland with the most commonly mentioned being:

▪ More public awareness (including through wider media presence and public health campaigns).
▪ Sustainable funding for regular support meetings, resources for rare disease charities/voluntary groups.
▪ More cross-border working to help build a local rare disease community similar to that which exists for persons living with cancer.
▪ Improved signposting to accredited existing information or the creation of new relevant information, ideally by the creation of a locally relevant online information hub.
▪ Development of a local rare disease registry that can integrate with international systems.
▪ A local rare disease coordinator.

One hundred percent of respondents were unable to satisfactorily obtain relevant information they were seeking about living with a rare disease(s) in Northern Ireland. Respondents highlighted needing more information that is easily accessible, particular for:

▪ More information for healthcare professionals that is easily accessible. GP surgeries in particular were specifically highlighted as needing more information to better manage rare disease patients.
▪ Information about sources of expertise—contact details for experts and services with mechanisms to access.
▪ Individual disease information from diagnosis to disease progression and treatment outcomes; links to best practice guidelines.

**Figure 6:**
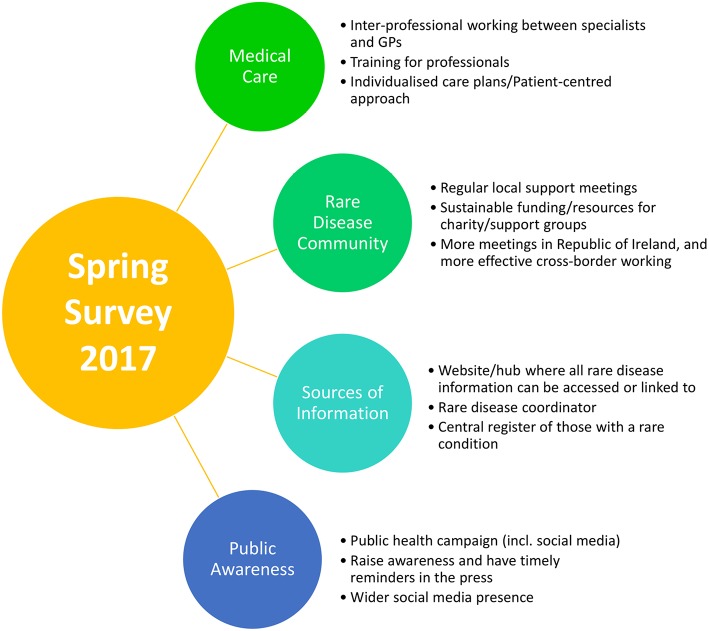
Priorities from thematic analysis of the information and communication survey results.

Overall, responses encompassed four overarching themes, illustrated in Figure 6 of priorities for change from individuals living and working with rare disease in Northern Ireland: improving medical care; signposting to reliable sources of information; building a rare disease community; and public awareness for rare disease(s).

### Literature Review

The literature search returned 308 papers resulting in the inclusion of 30 full text (Figure 1). With the addition of “Northern Ireland” in the search terms (i.e., “rare disease”[All Fields] AND “communication”[All Fields] AND “Northern Ireland”[All Fields]), no results were returned. Included articles were from Europe (the continent, Denmark, France, Germany, Italy, Malta, England, Spain, Sweden, and UK), Australia, and the United States of America (USA) (see Online Resource 2). Results from the survey aligned to internationally reporting outcomes from this literature review. This literature was categorized using the four themes, although themes 2 and 3, sources of information and rare disease community, overlapped considerably in the literature review results and so are included as a joint theme below.

## Discussion

### Context of Research

Communication can be especially challenging for those diagnosed with a rare condition with isolation described as an immediate consequence (5). Questions faced by many who are affected by a rare disease include: Due to the condition being rare, who can empathize with persons living or working with the condition? Who will be able to offer suggestions or experience? And who will have accurate or reputable information?

In a heavily technology dependent society, the internet now provides access to information in a way unimaginable 50 years ago. Therefore, it is necessary to take account of technology solutions for accessing rare disease information and improving communication mechanisms around rare disease. Isolation is a consistently reported theme (6) and the internet offers a practical solution in rural areas. However, even in Northern Ireland, approximately 56,000 premises do not have access to broadband internet and therefore cannot easily browse the web, access email, or use video services (7). Ofcom (7) reported that <1% of urban properties cannot get decent broadband compared to 23% of rural properties in Northern Ireland. Mazzucato et al. (8) demonstrated that the existence of a telephone helpline is one of the greatest measures of success in regards the evaluation of a national strategy around rare disease. Communication methods for persons living and working with a rare condition do need to be considered carefully to ensure equity of access to good communication and information tools.

Communication around rare disease is a challenge for researchers and healthcare professionals. Progress in the understanding of inherited rare diseases is often derived from international genetic research, in separate institutions globally and stored in differing formats. Local consolidated rare disease research infrastructures would facilitate effective global communication for rare diseases. International collaborations like IRDiRC (9) have made significant strides understanding rare disease mechanisms, but it is challenging for many countries to join such endeavors.

### Theme 1: Medical Care

#### Health and Social Care Professionals

Survey respondents revealed serious concerns about health and social care professionals not listening to patients or carers. Anonymous (10) states problems for parents speaking to geneticists include, being patronized, their research not being taken seriously, and a lack of empathy. Medical professionals do not ordinarily get the opportunity to explore how someone with a rare disease copes with day-to-day life yet Jeppeson et al. (11) state this valuable insight would allow for deeper understanding and more sympathetic communication. Greulich et al. (12) highlighted the lack of knowledge of general practitioners and suggest a web-based module might increase their knowledge of the specific disease and improve patient care. Budych et al. (13) have shown improved outcomes result from care providers recognizing and acknowledging patient's expertise, and be willing to partner with them in the decision making around treatment.

Our findings reported the internet as the most regularly accessed and perceived as the easiest approach to source information on rare disease. While some individuals regularly attend international conferences and trawl professional information sources to identify the latest research and treatment options for their rare disease(s), many people living with a rare disease describe accessing information *via* a general search of the internet as they find it considerably harder to access medical professionals, local and international information events and conferences, and the medical literature. This leaves the question of what affect does this have on the engagement between patient and medical professionals, and the patients' well-being who is relying on sourcing information themselves with no obvious credibility or reliability?

#### Diagnosis

A significant proportion of individuals found information around diagnosis and prognosis challenging. Better signposting to existing accredited health and social care information or the creation of relevant required information is needed. Post-diagnosis, information is also needed as a diagnosed patient may receive little or no information on what the short, medium, and long-term steps within the healthcare system are. Although a small study, Capioppo et al. (14) showed that parents' expectations on seeking diagnosis and information about their child's condition can be broken down into five categories; predictability, management of the condition, family planning, finding answers, and helping science.

#### Multidisciplinary Approach

The multidisciplinary approach can be challenging, as highlighted in a USA survey by McClain et al. (15) which evaluated communication systems for healthcare professionals dealing with rare disease. Berlage et al. (16), in the case of a patient with obstetric issues, illustrated how necessary or missed steps in a system could be identified, thus supporting the multidisciplinary approach. Further technology allows various healthcare professionals to collaborate to improve the welfare and care of patients (17).

A strategy of specified (co-located) appointments for rare disease patients post-diagnosis would facilitate more forward planning and cohesion between the specialities and aid a multidisciplinary approach. Although not directly addressed in any one question, many respondents commented that communication between services in the UK National Health Service (NHS) does not typically happen successfully for rare disease patients. For example, a respondent would like access to a “*care plan co-produced by a multidisciplinary team with the patient/carer at the centre*” and “*A co-ordinated and transparent approach at a Departmental level working with patients/carers/advocates to ensure a fully considered strategy is developed and implemented*”; and another “*There is complete unorganisation within the health service. No one wants to accept responsibility and information gets lost… They need to take a step back, sing from the same hymn sheet and realize that the person on the other end of the phone is going through life shattering changes.”*

In Europe established centers of expertise can specialize in one or a number of rare diseases, they include patients and their function is evaluated. As Taruscio et al. (18) state, it “*gathers or coordinates … multidisciplinary competences/skills, including paramedical skills and social services, in order to serve the specific medical, rehabilitative, palliative and social needs of RD (rare disease) patients… Centres of excellence should offer a wide range of specialized services: consultations, medical examinations, using specialized equipment, genetic testing, counseling and social care.”* Our survey indicates that this kind of accessible, all-in-one, source of information and support is an important goal for patients with a rare disease. Ideally Northern Ireland should develop a reference network hub as a unique access point to national and international centers of excellence, additionally acting as a communication network point between medical professionals.

#### Cross-Border Collaboration

Collaboration in the healthcare approach to rare disease of Northern Ireland and the Republic of Ireland has been identified as important (4). Although there can be challenges due to the different states involved, the current 24 European Reference Networks (ERNs) that have been established demonstrate that these challenges can be overcome (19). There is great capacity for cross-border collaboration and some legalization is already in place whereby, facilitated by ERNs, patients can access healthcare from other countries in the EU if they cannot get access to the care they need in their own country (20).

An example of effective cross-border care is between Malta and the UK, where Salbia et al. (21) identified four key components as supportive of the collaboration: “longevity and personal relationships” where medical professionals developed relationships to facilitate ease of the process; “communication and data sharing” facilitated by the UK receiving detailed patient summaries, results from investigations, and actions taken; a “shared care approach” where the UK teams treat the Maltese patients equitably, and accurate and timely information is sent back to the Maltese so that they can provide continuity of care for the patient; and “well-established support systems” such as transport to and from the hospital, accommodation for carers, and a contact for such issues (22).

International expert multidisciplinary clinics require capacity for their expertise to be shared with local healthcare providers, who may have patients with mobility limitations that cannot attend a centralized center. Work by Moreo et al. (22) on rare pulmonary diseases highlighted mobility limitations and that often family are heavily involved in the patients care, as in the cases of rare disease. They recommended providing training and education to the patient and their caregiver to aid with managing medication and to share the plan for care when transitioning between care services.

### Theme 2 and 3: Sources of Information and Rare Disease Community

Two responses suggested they had sufficient information, but this was a minority view, which raises the question, *are some rare diseases better dealt with in the NHS than other rare diseases?* Or is it the case that some healthcare providers are more proficient or successful in accessing this information on behalf of their patients, or at least pointing them in the right direction?

#### Support Groups

Half of our respondents accessed charity/support groups regularly; with charity support groups the second most regularly accessed source of information after the internet. Pauer et al. (23) affirms our findings that support groups provide invaluable information to those who are affected by rare disease. However, specialist support groups exist for only a minority of rare disease diagnoses. Further they found information such as genetic counseling to be lacking, similar to comments in our survey. Ultimately there is insufficient good quality assured information available and improvements around sources of information for those affected by rare disease is needed.

Doyle (24) highlights support groups are particularly important during the “transition” phase from childhood to adulthood were different age groups have different reflections on the interactions between themselves and others with their disease. Having support from the rare disease communities is invaluable, however having support from peers who are of a similar age and have been moving through the healthcare system at the same time as a patient is also important.

The issue of public stigma emerged from some responses to our survey. Zhu et al. (25) highlighted that groups of patients who are affected by the same disease empower one another to not be embarrassed by what is happening to them, and in such a way that they have confidence to advocate on behalf of others with their condition. Again highlighting the importance of support groups.

As Vicari and Cappai (26) found in exploring how connections are made *via* online methods, patient organization websites were more likely to connect people from their website to community formed pages (where groups of people affected by rare disease are communicating and sharing their own personally gained knowledge and experience), than they were to have people connect to other specific rare disease websites. Rocha et al. (27) reported patients increasingly using social media to seek information about diagnosis, peer support, and to read posts from rare disease organizations.

#### Psychological Support and Counseling

Challenges around accessing the right healthcare information and support were highlighted by our survey and Anderson et al. (28) who conducted a survey among the parents of children who had been diagnosed with a rare condition and referred to a specialist center. Similar to our own findings, and a very common theme, each family had varying experiences, some with very bad experiences in receiving diagnosis, often not being offered counseling, but others who received very good care from their general practitioners' who were very empathetic. The families reported a desire to access to their healthcare records, to make transitioning through their healthcare system much easier, and they valued support networks and wanted more information about social and psychological support.

#### Medical Records/Treatment

Providing easily accessible information on drug/treatment trials for rare diseases would be helpful for patients and healthcare professionals. Several rare disease drugs have been funded in Northern Ireland under managed access agreements; respondents are keen to know more about the process of obtaining orphan drugs. Stanarević Katavić (29) conducted semi-structured interviews with 15 respondents having one of three rare diseases, confirming that common challenges rare disease patients face include inaccessible new knowledge and lack of information about drugs and insufficient support from healthcare professionals.

Rare disease patients often see multiple healthcare professionals who may be based in different geographical locations and use different electronic systems. That creates challenges when a consultant is not aware of interactions with a patient and another medical professional; providing patients access to their own medical records such as tests, medical history, and communications from medical professionals would help them coordinate their rare disease care across medical specialties. Such resources exist for common conditions such as diabetes (30) or using tools such as PatientView (31). Ultimately the inclusion of a healthcare practitioner in the receipt of genetic information would be ideal, and the opportunity for the recipient to be able to get further tests and have access to reliable information is crucial (32).

#### Registries

From the respondents of the survey a desire for a rare disease registry was noted. Kourime et al. (33) state that key aspects in the design of registries are that they are patient based, have a sustainability strategy in place, have a group of people who manage the registry, that the information can be shared within the legal framework in place, and that it has sustainable funding. Recruitment of individuals to sign-up to a rare disease registry online was explored by Johnston et al. (34) who found that social media as the most effective, and cost-effective method of recruiting individuals, although there were demographic implications to this approach, such as a higher representation of women. Nevertheless they show that using social media platforms is a highly effective way of recruiting participants to an online registry and their methods could be replicable.

There are various types of rare disease registry in Europe: public health, clinical, genetic research and treatment registries (35), with differing methods of operating, and differing objectives. In order to make it possible for these registries to inter-work, homogeneity would need to be achieved in coding and diagnosis systems. Registries across Europe would be in favor of a portal through which the registries could all be accessed. However, they would also hope to gain from this other forms of support such as help with information technology, and shared resources (36).

#### Patient Advocacy Groups

Blay et al. (37) report patient advocacy groups are more open to information about current research questions and treatment strategies than the doctors. This compounds the problem of multidisciplinary working where even a reference network for rare cancers is not finding medical professionals to be willing participants in the progress and journey with the research community working on rare disease. Patient advocacy groups have also been found to be considerably useful when setting up a research network as they have direct communication with the people with a rare disease, and thus can more efficiently recruit people to a study, but should be utilized more frequently at the design stage of a study (38).

#### Other Concerns

Protection of personal information is not an isolated concern to those with rare condition, but it is of particular relevance. McCormack et al. (39) highlighted the concern around genetic discrimination due to the increasing progress in genetic research, were, although the desire for more access to clinical trials and cures was strong, there was concern that private companies could use the data negatively and mishandle that information. Gainotti et al. (40) point out that regular updates on how data is being used, and what research is achieving is valuable to the research participants. Further, inclusion of key rare disease organizations in governance decisions will promote a culture among the rare disease community of receptiveness to the benefits of research.

### Theme 4: Public Awareness

Public awareness (including through wider media presence and public health campaigns), was listed by 30% of the respondents as one of three priorities to improve information sharing and communication for rare disease(s) in Northern Ireland. A study by Castillo-Esparcia and López-Villafranca (41) investigated engagement of rare disease organizations with the public *via* media coverage and although a focus on human stories raised awareness, it did not increase knowledge of the public to specific diseases. Another study of online communication of rare disease organizations found that although most had websites, only a minority used their website to communicate with the media (42). Another study found media coverage being used as a tool to receive medication which had been denied previously through traditional means (43). The power of the use of the media here is clear—it can instigate action within health boards and systems, and within government.

Although the theme of public awareness produced fewer published papers, given the current era and trends a review of social media (rather than communication) and rare disease literature would more likely bring forth research that looking at rare disease focused public health campaigns or strategies. Such a review would be a valuable contribution, as public awareness is one of four overarching themes raised by our survey.

### Strengths and Limitations

An important strength of this study is that it is the first approach to gain views from a range of individuals living and working with rare diseases in Northern Ireland. The survey itself was developed in an iterative process by collaboration with a national rare disease charity (NIRDP). We appreciate that the approach promoting the survey primarily *via* the NIRDP may not have reached all individuals living and working with rare diseases in Northern Ireland, but it was selected as a pragmatic approach incorporating reach to more than 60 partner rare disease charities, medical professionals, patients, and carers. Social media has been used successfully to promote other health related surveys through charities and hospital groups [(44), p. 284 patient responses; (45), p. 155 responses]. Two hundred and forty individuals accessed this survey with responses demonstrating a variety of viewpoints from individuals self-selected as patients, family members of patients, carers, healthcare professionals, social support practitioners, policy makers etc. Of note, only 38% of respondents accessing the survey completed it in full, which may be partially explained by the nature of the question set; the survey was designed to collect important information without directing responses in free text fields, yet with as little respondent burden as possible. It was therefore possible to skip questions and those skipped tended to be around the question of promoting / barriers to engagement and suggesting three priorities for change. Further dissemination *via* print media and local radio may have helped generate more respondents for future research. There is no coordinated rare disease research infrastructure in Northern Ireland so the creation of a network for rare diseases in Northern Ireland, as proposed in this study, would further help improve dissemination and responses for future research.

Another strength of this study the mixed methods approach using an initial survey to evaluate the perceived availability of resources and communication preferences for rare diseases across Northern Ireland, followed by a literature review to place these findings in an international context. We appreciate that not everyone has access to or routinely uses the internet so the use of a primarily online resource may limit their participation. However, the survey was also available in hard copy and discussed at public meetings focused on rare diseases to be as inclusive as practically possible with available resources. Within the literature review, a limitation is that we only included studies in English due to the native language of the authors. We also restricted the formal literature review search strategy to articles published from 2012–2018 as a major aim was to place current findings from the survey within Northern Ireland in a wider context. Of note, this includes literature published since the launch of the UK strategy for rare disease (3) and the Northern Ireland Rare Disease Implementation Plan (4). Further 2018 & 2019 publications have been included to help discuss our results in the light of more recent knowledge. Widening the online resources searched and including other literature may have revealed further relevant information, however this was not planned as a systematic review and our approach does provide good coverage of the available relevant literature. Similarly, our search strategy was limited to the broad scope of “rare disease,” so we may have missed developments that are specific to a particular rare disease or more general rare disease terms.

## Conclusions

Many outcomes from our information and communication survey conducted in Northern Ireland mirror those from international research worldwide, emphasizing that much work remains to be conducted to improve communication around rare diseases. This research provides a wealth of consensus evidence that improvements to communication around rare disease are essential to improve the quality of life of those affected by a rare disease(s). The research also demonstrates the practical development of priorities for improvement based on the voices of those living and working with a rare condition.

With research recognizing the challenges faced by those affected by a rare condition, international collaborations are being formed that will bring significant improvements in health and social care systems. As changes are implemented, it is essential that further research be conducted to evaluate effectiveness and to ensure that the best possible structures and mechanisms are put in place to improve communication and information systems for those affected by a rare condition.

## Data Availability

The datasets used and/or analyzed during the current study are available from the corresponding author on request.

## Ethics Statement

This study is part of an information and communications review informing delivery of commitments in the Northern Ireland Rare Disease Implementation Plan. Informed consent was explicitly given by all participants by actively ticking a consent box before they could proceed with the online survey and this study has approval from research ethics committee, Northern Ireland.

## Author Contributions

AM conceived the project, collected the survey data, analyzed the data, contributed to data interpretation, and revised the manuscript following review. HM conceived the project, analyzed the data, and contributed to data interpretation. AC conducted the literature search, review, analyzed the survey data, and contributed to data interpretation. All authors contributed to drafting the manuscript and all authors agreed the final version for submission.

### Conflict of Interest Statement

The authors declare that the research was conducted in the absence of any commercial or financial relationships that could be construed as a potential conflict of interest.

## Abbreviations

ERNs: European Reference Networks
GP: General Practitioner
NHS: National Health Service
UK: United Kingdom
USA: United States of America.
