# Supplementary material for: Communication Needs for Individuals With Rare Diseases Within and Around the Healthcare System of Northern Ireland
Source: Front Public Health. 2019 Aug 21;7:236. doi: 10.3389/fpubh.2019.00236 (PMC6712370; doi:10.3389/fpubh.2019.00236)
Supplement: Supplementary file 1 [file Data_Sheet_1.PDF]

Title: Communication needs for individuals with rare diseases within and around the healthcare system of Northern Ireland.

Online Resource 1: Information and communication survey questions

1. How regularly did you access the following sources of information for rare disease(s)? Please select all that apply.

		**Regularly**	**Sometimes**	**Never**	**Unaware it existed**	**Would like to access more**
1	Local conference / information event					
2	International conference / information event					
3	Medical literature (eg. PubMed)					
4	Medical professional					
5	Non-medical professional					
6	Rare disease website (e.g. Orphanet)					
7	Charity / support group					
8	Television / radio / newspapers / magazines					
9	Family, friends, colleagues					
10	Social media					
11	Internet					
12	Other - please specify					

2. How easy did you find accessing the following sources of information for rare disease(s)? Please tick all that apply

**Answer Choice**		**Very easy**	**Manageable**	**Difficult**	**Impossible**	**Unaware it existed**
1	Local conference / information event					
2	International conference / information event					
3	Medical literature (eg. PubMed)					
4	Medical professional					
5	Non-medical professional					
6	Rare disease website (e.g. Orphanet)					
7	Charity / support group					
8	Television / radio / newspapers / magazines					

9	Family, friends, colleagues					
10	Social media					
11	Internet					
12	Other - please specify in comment box					

3. How would you prefer to access clinical (including research) information - please rank.

	Please Rank<br>(1-6, 1 highest, 6 lowest)
Charity or support group,	
Single individual or organisation coordinating information sources,	
Northern Ireland information hub (website) dedicated to rare diseases,	
Single telephone number for rare diseases,	
Variety of information sources and contact points,	
Other (please specify)<br>..	

4. What clinical information would you like to have available about rare disease(s)? (For example, diagnosis, symptoms, complications, treatment options, prognosis, research) Free text response

5. Please tell us about what non-clinical support you have accessed; please select all that apply

Financial (for example, benefits / grants)
Financial (for example, debt advice)
Legal (for example, discrimination)
Education (accessing, assessments, etc)
Family support
Housing
Peer support (connecting with others with similar challenges)

Non-clinical psychological support / counselling
Advocacy
Respite
Employment
Neurological care advisors
Other (please specify)<br>..

6. In your experience, what promotes good engagement with individuals / teams involved with rare disease(s)? (answers limited to 2,000 characters)
   Free text response

7. In your experience, what are the main barriers for good engagement with individuals / teams involved with rare disease(s)? (answers limited to 2,000 characters)
   Free text response

8. What do you think are the top three priorities to improve information sharing and communication for rare disease(s) in Northern Ireland? Free text within:

Priority 1	
Priority 2	
Priority 3	
Any further comments	

9. Have you tried to find information about living with a rare disease(s) in Northern Ireland and been unable to do so satisfactorily? Yes No

10. Are there sources of information you would suggest should be provided or made more accessible? Please tell us about the top three areas where you would like more information that is easily accessible. Free text within:

Area 1	
Area 2	
Area 3	
Any further comments	
