# Supplementary material for: Communication Needs for Individuals With Rare Diseases Within and Around the Healthcare System of Northern Ireland
Source: Front Public Health. 2019 Aug 21;7:236. doi: 10.3389/fpubh.2019.00236 (PMC6712370; doi:10.3389/fpubh.2019.00236)
Supplement: Supplementary file 2 [file Data_Sheet_2.PDF]

Title: Communication needs for individuals with rare diseases within and around the healthcare system of Northern Ireland.

Online Resource 2: Summary of articles resulting from literature search

**Authors**	**Title**	**Journal/ Book**	**Country of research**	**Main finding**
Anderson M, et al (2013)	Australian families living with rare disease: experiences of diagnosis, health services use and needs for psychosocial support.	Orphanet Journal of Rare Diseases	Australia	A survey was conducted among the parents of children who had been diagnosed with a rare condition and referred to a specialist centre. Each family had a varying experience, some with very bad experiences in receiving diagnosis, often not being offered counselling, but others who received very good care from their GP's who were very empathetic. The parents also would value access to their healthcare records and think this would make transitioning through their healthcare system much easier. They also valued support networks and would like to be given more information about social and psychological support.
Anonymous (2015)	Respectful communication by geneticists important for parents of children with undiagnosed disorders	American Journal of Medical Genetics	USA	Issues which were found by parents of children with undiagnosed disorders while communicating with the medical professionals were: being patronised; not having the research they'd done taken seriously; a lack of empathy.
Bañón Hernández AM, et al (2016)	The debate on rare diseases. A look at media response - Annual Review	Mètode Science Studies Journal	Spain	In this paper a particular case is highlighted where a family in Madrid used the media as a tool to receive medication for their son who had been denied twice through traditional means. The power of the use of the media here is clear – it can instigate action within health boards and systems, and within government.
Berlage S, et al (2015)	GerOSS (German obstetric surveillance system): A project to improve the treatment of obstetric rare diseases and complications using a web based documentation and information platform	Methods of Information in Medicine	Germany	Gave insight into how input of a patients journey into a data system allowed medical professionals to use it as a guide for future patients. It meant that necessary or missed steps could be identified, or symptoms and signs were recognised as important signals to how the situation will progress.
Blay J-Y, et al (2016)	The value of research collaborations and consortia in rare cancers	The Lancet Oncology	France	Had a valuable note on how information about current research questions and treatment strategies are far easier passed onto patient advocacy groups than the doctors themselves.
Budych K, et al (2012)	How do patients with rare diseases experience the medical	Health Policy	Germany	This directly looks at how the patients and the medical professionals interact –

	encounter? Exploring role behavior and its impact on patient-physician interaction			and how this is different from the normal patient-doctor interaction because in this scenario often the patient is the expert in regards their condition.
Cacioppo CN, et al (2016)	Expectation versus reality: the impact of utility on emotional outcomes after returning individualized genetic research results in pediatric rare disease research, a qualitative interview study.	PLoS One	USA	Explored here are the expectations of parents who were seeking diagnosis and information about their child’s condition. Parents expectations can be broken down into five categories; predictability, management of the condition, family planning, finding answers, and helping science.
Castillo-Esparcia A, et al (2015)	Online communication of patients with rare diseases in Spain	Revista Latina de Comunicación Social	Spain	The online communication of the organisations who are represented by people affected by rare disease was examined. It was found that most had websites, although not all, but that only a minority used their website to communicate with the media. Also only 3% of the websites were accessible for people with disabilities. Also those organisations who have a blog do not necessarily link the blog to their website which means there is a loss of traffic to both.
Castillo-Esparcia A, et al (2016)	Communication strategies employed by rare disease patient organizations in Spain	Ciência & Saúde Coletiva	Spain	Increased media coverage and attempted engagement of rare disease organisations with the public affected their visibility during the rare disease year in Spain in 2013. What was found was that the media focused on human stories and so although this raised awareness of rare diseases in general did not increase knowledge of the public in regards specific diseases. The rare disease organisations in Spain found that they did not receive more donations but did benefit from increased membership due to their campaigns and the increased press and media coverage.
Doyle M (2014)	Peer support and mentorship in a US rare disease community: findings from the Cystinosis in emerging adulthood study	Patient	USA	It is shown that having access to people who are experiencing similar problems or situations is something valued by the rare disease community, it is shown that this is very much the case for those with a rare disease who are moving from childhood to adulthood. Notably the different age groups have different reflections on the interactions between themselves and others with their disease. Sometimes those emerging into adulthood see those who’ve gone before as heroes, and sometimes see them as examples of how not to deal with the disease. This can be challenging for those in the community who didn’t have access to the latest treatments when they were diagnosed, or haven’t strictly adhered to their treatment regime.

Gainotti S, et al (2016)	Improving the informed consent process in international collaborative rare disease research: effective consent for effective research.	European Journal of Human Genetics	Europe	The consent process in regards information is addressed here, and is specifically in relation to collaboration internationally. It seems valuable to the research participants to have regular updates on how their data is being used, and what their research is achieving. And also to keep rare disease organisations included in governance decisions as they will promote a culture among the rare disease community of receptiveness to the benefits of research.
Greulich T, et al (2013)	Alpha1-antitrypsin deficiency - diagnostic testing and disease awareness in Germany and Italy	Respiratory Medicine	Germany and Italy	This article was very specific to one class of rare disease, but did highlight the lack of knowledge of general practitioners around it, and that even when guidelines are in place in regards the disease they are often not followed by them. What came out of the research was the indication that the medical professionals would appreciate more direct learning in regards the disease.
Héon-Klin V (2017)	European Reference Networks for rare diseases: what is the conceptual framework?	Orphanet Journal of Rare Diseases	Europe	It is being found in Europe that as European Reference Networks are being launched there are challenges to be overcome by the different states who are involved. There is also a recognised need for 'core players' within the networks, and so similarly here there would need to be a core group of people who mediate between different specialists and departments to ensure aims and targets are met which make the collaboration worthwhile.
Jeppesen J, et al (2014)	How narrative journalistic stories can communicate the individual's challenges of daily living with amyotrophic lateral sclerosis	Patient	Denmark	Medical professionals do not ordinarily get the opportunity to explore how someone with a rare disease goes about and copes with day to day life. When medical professionals do get this opportunity it was shown to be very valuable to the patient as it allowed for deeper understanding and more sympathetic communication.
Johnson K, et al (2014)	Evaluation of participant recruitment methods to a rare disease online registry	American Journal of Medical Genetics, Part A	USA	The authors found that social media was the most effective, and cost effective, method of recruiting individuals to a rare diseases registry. However there were demographic implications of using this approach, such as the higher representation of woman.
Kourime M, et al (2017)	An assessment of the quality of the I-DSD and the I-CAH registries -international registries for rare conditions affecting sex development	Orphanet Journal of Rare Diseases	Europe	In the design of a registry the views and needs of each set of stakeholders is very important in order for each user to be able to get out of the registry what they need to further the development of rare disease information. Some key aspects which make a registry truly stand out are that they are patient based, have a sustainability strategy in place, have a group of people who do and will manage

				the registry, that the information can be shared within the legal framework in place, and that it has funding.
McClain MR, et al (2014)	A survey of the preferences of primary care physicians regarding the comanagement with specialists of children with rare or complex conditions.	Clinical Pediatrics	USA	Notably from this research was the finding that for the family practitioners, and the paediatricians, the most valuable information that they receive about a child with complex issues are: firstly an action plan for the patient which specifies who is to take what action and when they are to do it; secondly an emergency care plan written for them to follow if necessary; thirdly a letter written by the specialist which will include plans, findings from the consultation, and actions taken; and finally a fact sheet with key information about the patients' condition.
McCormack P, et al (2016)	You should at least ask'. The expectations, hopes and fears of rare disease patients on large-scale data and biomaterial sharing for genomics research.	European Journal of Human Genetics	England	Concern around genetic discrimination due the increasing progress in genetic research was addressed. In this case although the desire for more access to clinical trials and cures was strong there is concern that if a database was set up with too much free access that private companies could use the data gained from individuals negatively and that their information could be mishandled.
Merkel P, et al (2016)	The partnership of patient advocacy groups and clinical investigators in the rare diseases clinical research network	Orphanet Journal of Rare Diseases	USA	Patient advocacy groups are of considerable use when setting up a research network. However from this paper one of the challenges for the patient advocacy groups is their capacity to have an input into how a study in the research network will be designed. However they did value getting to attend meetings in relation to research and being included in communications such as conference calls.
Moliner MA, et al (2017)	The European Union policy in the field of rare diseases	Rare Diseases Epidemiology: Update and Overview.	Europe	Policy around rare diseases has many factors to take into consideration. In Europe there is great capacity for cross-border collaboration and some legalisation is already in place whereby patients can access healthcare from other countries in the EU if they can't get access to the care they need in their own country. European reference networks are crucial in facilitating this process. Patient organisations have also been invaluable in regards forming rare disease policies and in aiding with collaborative projects.
Moreo K, et al (2017)	Integrated transitions of care for patients with rare pulmonary diseases	Professional Case Management	USA	It is important that there is the capacity for the expertise held within a centralised centre to be shared with local healthcare providers who may be trying to meet the needs of someone who is not able to attend a centre due to mobility limitations. They also noted that as in the cases of rare disease often family are

				heavily involved in the patients care, and that it is ideal to provide training and education to the patient and their caregiver to aid with managing medication but also to provide the plan for care when transitioning between care services.
Oliveri S, et al (2016)	Let the individuals directly concerned decide: A solution to tragic choices in genetic risk information	Public Health Genomics	Sweden	It was found here that the inclusion of a healthcare practitioner in the receipt of genetic information would be ideal, and the opportunity for the recipient to be able to get further tests and have access to reliable information is crucial.
Pauer F, et al (2017)	Rare diseases on the internet: an assessment of the quality of online information.	Journal of Medical Internet Research	Germany	Support groups for those with rare disease provide invaluable information to those who are affected by rare disease, and there is not often better information found elsewhere. Ultimately there is not enough good quality assured information out there and improvements around sources of information for those affected by rare disease need to be made.
Saliba V, et al (2014)	Clinicians', policy makers' and patients' views of pediatric cross-border care between Malta and the UK	Journal of Health Services Research & Policy	Malta/UK	Four key facts identified in this study as supportive of the collaboration between the UK and Malta were: 'longevity and personal relationships' where medical professionals developed relationships to facilitate ease of the process; 'communication and data sharing' facilitated by the fact that although full medical records aren't available to the UK medical professionals they are sent detailed patient summaries and results from investigations or actions that have been taken with the patient and the Maltese medical professionals; a 'shared care approach' where the UK teams treat the Maltese patients as they would anyone referred from another centre in the UK; and there are 'well established support systems' where practical concerns are taken care of such as transport to and from the hospital, accommodation for carers, and that in this they have a person who they can reliably contact.
Santoro M, et al (2015)	Rare disease registries classification and characterization: a data mining approach.	Public Health Genomics	Europe	Registries have differing methods of operating, and in regards improvement are not seeking after the same main objectives. In order to make it possible for these registries to inter-work homogeneity would need to be achieved in coding and diagnosis systems.
Taruscio D, et al (2013)	The current situation and needs of rare disease registries in Europe	Public Health Genomics	Europe	Registries across Europe would be in favour of a portal through which the registries could all be accessed. However they would also hope to gain from this other forms of support such as help with information technology, and shared

				resources.
Taruscio D, et al (2014)	Centres of expertise and European Reference Networks: key issues in the field of rare diseases. The EUCERD recommendations	Blood Transfusion	Europe	This paper effectively demonstrates what the concept of a Centre of Excellence is in Europe, and what a Centre of Excellence hopes to deliver for people with rare diseases. Interestingly they note that it is important in the concept stage of the Centre of Excellence to designate their function. This function should be evaluated and the evaluation process should include patients.
Teixeira L, et al (2012)	Improvement of surveillance of hemophilia treatment through ICTs	IEEE	USA	Shows that technology can be made accessible to various healthcare professionals and that they can collaborate using this to improve the welfare and care of the patient.
Vicari S, et al (2016)	Health activism and the logic of connective action. A case study of rare disease patient organisations	Information Communication and Society	Italy	Here it is shown that connections can be made via online methods, and how rare disease patient organisations use the online platforms to communicate with each other and with the public. It was found that patient organisation websites connected people from their website to community formed pages, where groups of people affected by rare disease are communicating and sharing their own personally gained knowledge and experience. This connection was more likely to be made than to have people from their website connect to other specific rare disease websites.
Zhu X, et al (2017)	Living with a rare health condition: the influence of a support community and public stigma on communication, stress, and available support	Journal of Applied Communication Research	USA	In this paper it is highlighted that the groups of patients who are affected by the same disease empower one another to not be embarrassed by what is happening to them, and empower them in such a way that they have confidence to advocate on behalf of others with their condition. Thus this paper highlights the importance of having support groups for those affected by rare disease.
